# In Vivo and In Vitro Effects of Fermentable Dietary Fiber from High-Amylose Wheat Containing Resistant Starch on the Intestinal Environment: A Randomized, Double-Blind, Placebo-Controlled, Human Trial

**DOI:** 10.3390/microorganisms14040797

**Published:** 2026-04-01

**Authors:** Ryo Iwata, Yuto Otomo, Yasuyuki Nishitsuji, Junichi Node, Kazuki Toyota, Shukuko Ebihara, Yosuke Kikuchi

**Affiliations:** 1 Nisshin Seifun Group Inc., 5-3-1 Tsurugaoka, Fujimino-City 356-8511, Saitama, Japan; iwata.ryo@nisshin.com (R.I.); otomo.yuto@nisshin.com (Y.O.); nishitsuji.yasuyuki@nisshin.com (Y.N.); node.junichi@nisshin.com (J.N.); toyota.kazuki@nisshin.com (K.T.); 2 Chiyoda Paramedical Care Clinic, 3-3-10 Hongokucho, Chuo-ku, Tokyo 103-0021, Japan; s.e@cpcc.co.jp

**Keywords:** high-amylose wheat, resistant starch, intestinal function, bowel movements, short-chain fatty acid, butyrate, p-cresol, randomized controlled trial, fermentable dietary fiber

## Abstract

This study investigated the effects of fermentable dietary fiber derived from high-amylose wheat (HAW) flour on the intestinal environment using an in vitro fecal fermentation assay and a randomized, double-blind, parallel-group clinical trial. Digested HAW flour was fractionated into total dietary fiber (TDF), resistant starch (RS), and non-RS dietary fiber (DF-RS) fractions. Fecal culture tests were used to quantify short-chain fatty acid (SCFA) production and microbiota composition after cultivation. In the randomized, double-blind, parallel-group trial, 76 healthy adults consumed HAW-containing food (dietary fiber: 5.5 g/day, RS: 2.9 g/day) or control food (dietary fiber: 0.7 g/day, RS: n.d.) for 2 weeks. Both RS and DF-RS increased SCFA production, with TDF having even stronger effects, suggesting enhanced fermentability in the presence of multiple types of fermentable dietary fibers. In the human trial, HAW-containing food intake did not significantly alter bowel movement frequency compared with the control. However, HAW-containing food consumption significantly reduced the levels of p-cresol, a representative gut-derived proteolytic metabolite linked to intestinal dysbiosis. No significant differences were observed in other secondary endpoints. Intake of HAW-derived foods appears to promote SCFA production and improve the intestinal environment by reducing p-cresol accumulation. Overall, these results highlight HAW flour as a practical prebiotic ingredient that helps support gut health.

## 1. Introduction

Accumulating evidence has demonstrated the close association of the intestinal environment with the maintenance of health and prevention of various diseases [1,2]. In addition to its roles in the digestion and absorption of dietary components, the intestine participates in essential physiological functions, such as promoting bowel movements through peristalsis, protecting against pathogens and toxins via epithelial barrier function, and producing essential nutrients, including vitamins, through the intestinal microbiota [3,4,5]. Disruption of the intestinal environment has been linked to increased risks of various diseases, including inflammatory bowel disease, obesity, dementia, and depression. Therefore, continued maintenance of intestinal health is considered crucial [6,7].

The diet is a major factor influencing the intestinal environment. For instance, high-protein or high-fat diet consumption has been reported to induce intestinal dysbiosis and stimulate the intestinal production of putrefactive metabolites [8,9]. Conversely, high dietary fiber intake improves microbial diversity and increases the levels of beneficial metabolites, such as short-chain fatty acids (SCFAs) [10]. Arabinoxylan derived from wheat bran increases the abundance of butyrate-producing bacteria and consequently enhances butyrate levels in the intestine [11], and potato-derived resistant starch (RS) consumption was found to increase bowel movement frequency through changes in the intestinal microbiota [12].

A high-amylose wheat (HAW) variety containing higher RS content than conventional wheat has recently been developed. This wheat variety exhibits reduced expression of the starch branching enzyme (SBE) II isoforms SBEIIa and SBEIIb, resulting in markedly elongated amylopectin side chains and thus increased proportions of an amylose-like amylopectin structure [13]. These differences explain the high RS content of this wheat variety in analyses based on the AOAC 2002.02 method [13,14].

Human trials using food containing HAW flour found that daily intake of 11.1 g of wheat-derived RS for 4 weeks increases fecal butyrate levels and the proportion of SCFA-producing bacteria while decreasing fecal p-cresol levels [15]. In another study, Japanese participants consumed 3.3 g/day of wheat-derived RS for 4 weeks, resulting in no significant improvement in bowel movements in the full statistical analysis; however, subgroup analysis of subjects experiencing strong sensations of incomplete evacuation confirmed an increase in bowel movement frequency [16].

Typical wheat flour contains little RS but includes dietary fibers such as arabinoxylan, arabinogalactan, and fructan [17]. These dietary fibers are metabolized by intestinal bacteria to produce SCFAs [18,19,20]. Meanwhile, HAW flour contains considerable amounts of these dietary fibers alongside RS. Thus, combining different types of dietary fibers could potentially have distinct effects on the intestinal microbiota and its metabolite production. For example, in vitro studies revealed that combinations of multiple dietary fibers, including arabinoxylan, RS, and β-glucan, enhance SCFA production and improve intestinal microbiota diversity [21].

Additionally, research on personalized nutrition, which aims to design diets tailored to individual intestinal environments, has attracted increasing attention in recent years, because of large individual differences [22]. For example, butyrate production varies significantly among individuals even with the same dietary fiber intake [23]. Such variability has been attributed to differences in individual intestinal environments, including insufficient intake of vitamin B1, which is essential for the growth of butyrate-producing bacteria, or low abundance of *Bifidobacterium*, which produces the butyrate precursor acetate. Therefore, understanding the characteristics of responders and non-responders to prebiotics intake is essential to propose optimal diets for individuals, especially from the perspective of personalized nutrition.

Nevertheless, the combined and overall effects of RS and other fermentable dietary fibers in HAW flour on the intestinal microbiota and their metabolites have not been fully elucidated. In addition, the characteristics of responders and non-responders to HAW intake remain unclear. Thus, to elucidate the overall effects of the fermentable dietary fibers in HAW flour, the impact of HAW flour intake on bowel movement frequency, intestinal microbiota, and bacterial metabolites was investigated through both culture tests and a human trial, including stratified analysis to clarify the characteristics of responders.

## 2. Materials and Methods

### 2.1. Human Fecal Microbiota Culture Assay

#### 2.1.1. Materials

One gram of HAW (product name: AMULEIA, Nisshin Seifun Co., Ltd., Tokyo, Japan) was added to 20 mL of pure water and stirred well. To mimic the cooking process of the flour, the mixture was placed in a constant temperature dryer (SSR-114S; Isuzu Seisakusho Co., Ltd., Sanjo, Niigata, Japan) set at 95 °C and heated for 30 min after the temperature of the liquid reached 95 °C, which was confirmed using a thermometer. After heating, the mixture was frozen and dried for 2 days using a freeze dryer (FDU-1200; Tokyo Rikakikai Co., Ltd., Tokyo, Japan) no longer changed. Consequently, 0.89 g of heated HAW flour with a final moisture content of 1.3% was obtained. The resultant powder was termed “heated HAW flour.”

Enzymatic dietary fiber material treatment mimicking digestion and absorption in the body was performed according to the AOAC 2011.25 method [24]. For 1.0 g of heated HAW flour, the Integrated Total Dietary Fiber Assay Kit (K-INTDF, Megazyme, Bray, Ireland) was used to recover the insoluble fraction and soluble high-molecular-weight fraction from the sample, which were then dried in a freeze dryer for 1 day. The filtrate containing the recovered soluble low-molecular-weight fraction was subjected to ethanol removal using a rotary evaporator (R-114; BUCHI Labortechnik AG, Flawil, Switzerland). The residue was redissolved in pure water and dialyzed for 24 h in tap water using a Spectra/Por CE dialysis membrane (MWCO 500–1000 Da; Funakoshi Co., Ltd., Tokyo, Japan) to remove monosaccharides and disaccharides. The resulting solution was dried using a freeze dryer for 3 days to obtain the soluble low-molecular-weight fraction. After all recovered fractions were combined, the final yield was 0.21 g. The combined fractions were designated as the total dietary fiber (TDF) fraction.

Additionally, enzymatic hydrolysis of starch, including resistant starch, was performed in accordance with AOAC 2001.03. Heated HAW flour (1.0 g) was added to a beaker and mixed with 50 mL of phosphate buffer (pH 6.0). Then, 50 μL of thermostable α-amylase (E-BSTAA, Megazyme, 3 KU/mL) was added. The beaker was covered with aluminum foil and heated for 30 min with stirring in an oil bath (EYELA PS-1000, Tokyo Rikakikai Co., Ltd., Tokyo, Japan) at 98–100 °C, with the speed controller set to level 5, to facilitate the breakdown of RS present in the HAW flour. Following incubation, the mixture was cooled to room temperature, and 10 mL of 0.275 N NaOH was added to adjust the pH to 7.5 ± 0.1, thereby inactivating the enzyme, following AOAC 2001.03. Subsequent procedures were performed in accordance with AOAC 2011.25. The final yield of the non-RS dietary fiber (DF-RS) was 0.10 g.

Additionally, 100 g of HAW flour was mixed with 80 mL of pure water using a versatile mixer (Dalton Co., Ltd., Tokyo, Japan) for 2 min to create dough. Next, 500 mL of pure water was added to elute the starch from the dough. The recovery of powdered starch from the liquid after starch elution was performed as described previously [25]. The obtained powder was subjected to the same heat treatment as the heated HAW flour. Additionally, 1.0 g of the obtained powder was processed according to AOAC 2011.25. The yield of the resistant starch (RS) fraction was 0.07 g.

In this study, total dietary fiber (TDF) was defined as the sum of resistant starch (RS) and the non-starch dietary fiber fraction (DF-RS), such that TDF = RS + DF-RS. Based on the amounts of each fraction in the HAW flour, TDF, RS, and DF-RS were added to the 500 μL medium at 0.3% (*w*/*v*), 0.105% (*w*/*v*), and 0.136% (*w*/*v*), respectively.

#### 2.1.2. Donors

Nine healthy adults with no significant medical history in the past 6 months provided fresh stool samples on the experimental day. The representativeness and biological diversity of the samples were evaluated by comparison with a large-scale Japanese gut microbiota dataset. Based on this assessment, the sample set was considered representative of the general Japanese population and sufficient in diversity for the purposes of this exploratory in vitro study. The sample size was determined based on current standard practices in in vitro fecal fermentation studies, in which donor cohorts of 3–10 individuals are commonly used to capture biologically relevant trends while maintaining experimental control [26,27]. Accordingly, nine independent donors were included in this study.

#### 2.1.3. Fermentation

The in vitro culture assay of human fecal microbiota (MGScreening™) was performed using a method developed by Metagen Inc. (Tsuruoka, Yamagata, Japan) [28]. Fresh fecal samples collected from nine healthy participants were used as inocula and added to the culture medium at 0.1% (*v*/*v*). The culture medium consisted of modified YCFA medium with inherent buffering capacity and was adjusted to an initial pH of 7.2. Fermentations were conducted in a total working volume of 500 μL per culture under static incubation. As test samples, a blank control and the samples TDF, RS, and DF-RS were added to the culture medium at final concentrations of 0.3% (*w*/*v*), 0.105% (*w*/*v*), and 0.136% (*w*/*v*), respectively. The test samples were cultured for 16 h under anaerobic conditions (H_2_: 2.0–2.2%, O_2_: 0 ppm) at 37 °C. Afterward, the fecal culture medium was centrifuged at 8000× *g* for 10 min at 4 °C. The obtained sediment was used for nucleic acid extraction for microbiota analysis, and the supernatant was used for metabolite extraction for metabolite analysis.

#### 2.1.4. DNA Extraction and Sequencing

DNA extraction was performed as previously reported [29]. Briefly, fecal samples were lyophilized, mechanically disrupted by bead-beating, and DNA was extracted using an automated extraction system (GENE PREP STAR PI-480; Kurabo Industries Ltd., Osaka, Japan) according to the manufacturer’s protocol. The V1–V2 region of the 16S rRNA gene was extracted using the universal primers 27F-mod (5′-AGRGTTTGATYMTGGCTCAG-3′) and 338R (5′-TGCTGCCTCCCGTAGGAGT-3′) and amplified using Tks Gflex DNA polymerase (Takara Bio Inc., Kusatsu, Shiga, Japan). The amplified products were sequenced using paired-end methods on a NextSeq 1000 (Illumina, San Diego, CA, USA) according to the manufacturer’s protocol. The average sequencing depth was 49,198 ± 1421 reads per sample, with a minimum of 37,098 reads.

#### 2.1.5. Metabolome Analysis

Metabolome analysis was performed to quantify SCFAs, including acetic acid, propionic acid, and butyric acid. SCFAs were extracted from the fecal culture medium as previously reported [29]. Briefly, a defined amount of fecal sample was mixed with an acidic aqueous solution containing an internal standard, followed by extraction with an organic solvent at a fixed sample-to-solvent ratio. After vigorous mixing and centrifugation, the organic phase was collected for analysis. The extracted SCFAs were chemically derivatized to improve volatility and chromatographic resolution and subsequently analyzed using GC–MS. For each compound, the column retention time, mass-to-charge ratio (*m*/*z*), and peak area were obtained. Individual SCFAs were identified by comparing their retention times and mass spectra with those of authentic reference standards analyzed under identical GC–MS conditions. Quantification was performed using external calibration curves prepared from serial dilutions of SCFA standard solutions. An internal standard was used to normalize peak areas, and SCFA concentrations were calculated based on the resulting calibration curves.

#### 2.1.6. Bioinformatics and Statistical Analysis

Microbiota analysis was performed following previously reported methods [30]. For 16S rRNA gene-based microbiome analysis, QIIME2 (version 2019.10) was used. To evaluate changes in gut bacteria among the groups, all statistical analyses were performed using a Python script (version 3.7.6) developed by Metagen Inc. The Wilcoxon signed-rank test was used to compare bacterial changes between the control and treatment groups. Bacteria with an average relative abundance smaller than 0.1% were excluded from the comparison. No adjustment was made for multiple testing of multiple items and multiple time points.

### 2.2. Clinical Trial

#### 2.2.1. Trial Design

This study adhered to the Declaration of Helsinki (approved in 1964, revised in October 2013) and followed the Ethical Guidelines for Life and Medical Sciences Research Involving Human Subjects (Ministry of Education, Culture, Sports, Science and Technology; Ministry of Health, Labor and Welfare; Ministry of Economy, Trade and Industry, Notification No. 1, partially revised on 27 March 2023). The scientific and ethical validity of the study was confirmed by the Ethics Review Committee of Chiyoda Paramedical Care Clinic (IRB No: 25051603, approval date 16 May 2025). The study was registered in the UMIN Clinical Trials Registration System (UMIN-CTR) (UMIN ID: UMIN000058690, registration date: 4 August 2025, https://center6.umin.ac.jp/cgi-open-bin/ctr/ctr_view.cgi?recptno=R000066211, accessed on 29 March 2026) and conducted from May 2025 to March 2026. This study comprised a randomized, double-blind, placebo-controlled, parallel-group comparison trial with an 8-week pre-observation period and a 2-week intake period, during which a trend toward increased bowel movement frequency was observed in a previous pilot study (UMIN: 000051359, unpublished).

#### 2.2.2. Eligibility Criteria

Study subjects were publicly recruited by CPCC Corporation (Tokyo, Japan) starting in May 2025. The participants comprised healthy men and women who voluntarily agreed to participate in the study and had difficulties related to bowel movement frequency. The inclusion criteria were as follows:

(1) healthy men and women aged 20 to less than 65 at the time of providing study consent;
(2) ability to consume one study food item per day and receive study food items each week; and
(3) agreement to participate in the study after receiving a sufficient explanation of the study and understanding the content.

Among those who provided written consent, individuals were selected according to a 2-week diary conducted as a preliminary survey, confirming that their bowel movement frequency was five times or less per week.

The exclusion criteria were as follows:

(1) consumption of functional health foods, foods with functional claims, health foods, or supplements that can affect the gut environment two or more times a week (individuals who cannot stop consuming these foods from the time of consent were excluded, even if consumption frequency was one or fewer times per week);
(2) consumption of yogurts, lactic acid bacteria-containing beverages, or staple foods rich in dietary fiber (e.g., brown rice, barley, whole-grain bread, cereals) not classified as functional health foods three or more times a week;
(3) alcohol consumption three or more times a month and consumption exceeding 60 g per occasion;
(4) consumption of one or zero meals at least 1 day a week from 1 month prior to the preliminary examination;
(5) practicing carbohydrate restriction or dieting;
(6) extremely irregular lifestyles (such as night shifts or irregular rotating shifts);
(7) BMI ≥ 30.0 kg/m^2^;
(8) plans to change existing lifestyle habits, dietary habits, or living environment (such as relocation, job transfer, overseas travel, or overseas business trips) during the study period;
(9) plans to participate in other clinical studies after agreeing to participate in this study;
(10) participation of other household members in the study;
(11) history of appendectomy;
(12) surgery (e.g., colonoscopic surgery, gallstone and gallbladder removal, gastric bypass surgery) that might affect the study outcomes within 6 months of providing consent;
(13) use of medication that might affect the intestinal environment (e.g., antibiotics, probiotics, laxatives, proton pump inhibitors) within 1 month prior to the preliminary screening or plans to use such medications during the study period;
(14) current oral treatment or dietary therapy;
(15) prior or current serious diseases affecting major organs (e.g., heart, liver, kidneys, digestive system);
(16) pregnancy, breastfeeding, or plans to become pregnant during the trial period;
(17) allergies to pharmaceuticals and foods (particularly wheat, eggs, milk, walnuts, and soybeans);
(18) donation of component blood or 200 mL of whole blood within 1 month prior to the start of the study;
(19) among men, donation of 400 mL of whole blood within 3 months prior to the start of the study;
(20) among women, donation of 400 mL of whole blood within 4 months prior to the start of the study;
(21) combined total blood collection within 12 months prior to the start of the study, plus the study’s planned total blood collection amount, exceeding 1200 mL in men;
(22) combined total blood collection within 12 months prior to the start of the study, plus the study’s planned total blood collection amount, exceeding 800 mL in women; and
(23) deemed unsuitable for study participation by the principal investigator or sub-investigator.

In accordance with the approved trial protocol, the objectives and content of the study were thoroughly explained to the subjects using documents approved by the ethics review committee, ensuring that subjects fully understood the information and consented of their own volition. Prior to the study’s implementation, all subjects were instructed concerning adherence to certain lifestyle guidelines and prohibited activities during the trial period. Subjects visited Chiyoda Paramedical Care Clinic, where they completed background surveys; interviews, physical measurements, brief self-administered diet history questionnaires (BDHQ); blood and urine tests before the intervention period; and interviews, physical measurements, blood, urine tests, and Constipation Assessment Scale—Modified (CAS-MT) before and after the consumption period. The follow-up period was determined as necessary by the principal investigator during the study period.

#### 2.2.3. Intervention

Two food products were used for the trial: HAW-containing and control foods. The HAW-containing food included HAW flour, margarine, sugar, chicken eggs, salt, and baking powder. Conversely, the control food contained common wheat starch (Wheat Starch Marutoku; Nagata Sangyo Co., Ltd., Shiso, Hyogo, Japan) and wheat protein (Superglue 85H; Nihon Colloid Co., Ltd., Tokyo, Japan) instead of HAW flour, ensuring no distinguishable differences in taste or appearance from the HAW food. Prior to the trial, a sensory comparison was conducted to confirm that the two food products were comparable in terms of taste, flavor, and appearance.

The assigned HAW-containing or control food was provided to the subjects in a frozen state and thawed on the day of consumption (approximately 40 s in a microwave at 500 or 600 W) to replace one main meal (breakfast, lunch, or dinner) per day. Compliance with the prescribed daily consumption was monitored using a daily intake diary and through brief interviews conducted at each study visit. The per-serving nutritional composition and dietary fiber content of the test and control foods are listed in Table 1.

#### 2.2.4. Outcomes

The effectiveness of the test food was evaluated using an intention-to-treat (ITT) analysis for subjects who completed test food intake and adhered to all study protocols. Efficacy was evaluated according to the criteria set in the trial protocol. The primary outcome was the bowel-regulating effect (frequency of bowel movements), as recorded in daily life diaries. Secondary evaluation items included total SCFA content (sum of acetic acid, propionic acid, and n-butyric acid content) in feces, individual SCFA (acetic acid, propionic acid, n-butyric acid) levels in feces, organic acid (iso-butyric acid, lactic acid, succinic acid, n-valeric acid, iso-valeric acid, 2-methylvaleric acid, 4-methylvaleric acid, caproic acid, malonic acid, formic acid) content in feces, moisture content in feces, fecal pH, bowel movement status (number of days with bowel movements, stool volume, stool consistency, stool color, feeling of residual stool at the time of defecation, odor), CAS-MT, intestinal microflora, and fecal spoilage product (phenol, 4-ethylphenol, p-cresol, indole, skatole) levels. Evaluations were performed before and after the consumption period. To protect subjects’ privacy and personal information, all test-related data were anonymized. No numbers that could identify the subject’s name or identity were applied, and a method specifying subjects through identification numbers was adopted.

Subjects were asked to complete a daily life diary for 10 weeks, including 8 weeks before the intervention and 2 weeks during the intervention. The bowel movement status recorded in the diary included the presence or absence of bowel movements, frequency of bowel movements, fecal volume (converted into number of eggs), fecal characteristics (1, hard pellets; 2, hard; 3, slightly hard; 4, normal; 5, slightly soft; 6, mud-like; 7, watery), fecal color (1, yellow; 2, ocher; 3 light brown, 4, brown; 5, dark brown; 6, black), residual sensation of feces during bowel movements (1, no sensation of remaining feces, refreshing and invigorating; 2, almost no sensation of remaining feces, refreshing; 3, slight sensation of remaining feces; 4, sensation of remaining feces, not refreshing), and odor (1, very weak; 2, weak; 3, normal; 4, strong; 5, very strong). The number of days with bowel movements and frequency of bowel movements were totaled weekly. The average value per defecation was calculated weekly. Additionally, the consumption status of test and control foods, changes in daily life (meal frequency, quantity, alcohol consumption, exercise amount, sleep duration, living environment), intake status of health foods and alcohol, self-recognized physical symptoms, medical institution visits, treatment details, medicine use, and vaccination status and type were also recorded in the life diary.

During the study, fecal samples were collected at baseline and after 2 weeks of test food consumption and frozen at −20 °C until intestinal microbiome and metabolome analyses, which were conducted at Metagen Inc. using the same methods as described in Section 2.1.4 and Section 2.1.5. Measurement of fecal putrefactive metabolites was performed using a slightly modified method previously reported [33]. The amount of SCFAs per bowel movement was calculated from the bowel movement volume recorded in the diary (converted into the number of eggs, with one egg of feces equivalent to 50 g). The fecal moisture value was set at 74.6% based on a previous study [34].

#### 2.2.5. Safety

In evaluating safety, the principal investigator considered all adverse symptoms or signs experienced by subjects during the intake period, as determined by interviews, physical measurements, physiological testing, hematology, blood biochemistry, infection testing, urinalysis, and diary entries during each clinic visit, as adverse events.

#### 2.2.6. Sample Size

The number of required participants was calculated using G*Power 3.1.9.7 (Heinrich Heine University Düsseldorf, Düsseldorf, Germany) based on the results of a previous pilot study, with a significance level of 5% and power of 0.95. A previous pilot study involving 40 participants evaluated the effects of HAW-derived RS on the intestinal microbiota in subjects having five or fewer bowel movements per week. The study employed a 12-week parallel-group design in which participants consumed the test food throughout the intervention period. At the start of observation, the mean number of weekly bowel movements was 4.4 ± 0.9 in the control group and 4.0 ± 1.0 in the HAW group. At this 2-week time point, the number had increased to 5.0 ± 1.7 in the control group, versus 6.4 ± 1.5 in the HAW group (UMIN: 000051359, unpublished). From these numbers, the required number of subjects was calculated as 72. The target number of subjects for this study was set at 76 to account for a potential 5% dropout rate during the study period.

#### 2.2.7. Randomization and Blinding

**Figure 1:**
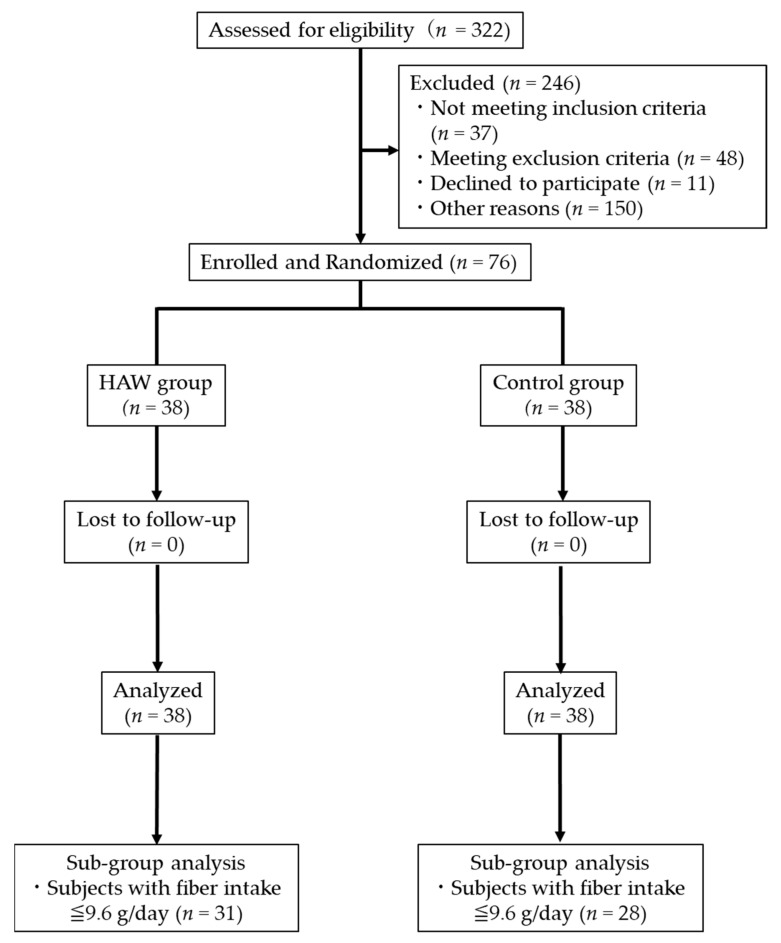
CONSORT 2025 flow diagram for study participants.

Figure 1 presents the flowchart of the study. The assignment order of subjects was created by CPCC, and 76 subjects who met the inclusion and exclusion criteria were enrolled by the principal investigator, with assignment conducted by Evidence Marketing LLC. Specifically, using stratified block randomization with adjusting factors such as age, sex, and bowel movement frequency in the diary for 2 weeks starting the day after pretesting, subjects were randomly assigned to the control or HAW-containing food group (38 subjects/group). The assignment table was sealed by the assignment manager and stored until opened. Furthermore, blinding integrity was maintained for the physicians conducting the test, subjects, medical facility staff, and all research-related personnel.

#### 2.2.8. Statistical Methods

To avoid arbitrary analysis, data considered outliers were slated for inclusion in the evaluation; however, no outliers arose in the efficacy or safety evaluations. If a measuring error was clearly causal, then the related data were excluded from the analysis. Additionally, missing values were not supplemented and were excluded from the analysis.

To compare characteristics between the groups, measurement values before and after intake and changes from pre-intake were displayed as the mean ± standard deviation. For items other than the intestinal microbiota, normality was assumed, Student’s *t*-test was used for analysis. For the intestinal microbiota, normality was not assumed, and the Mann–Whitney U test was used. For statistical analysis, Excel for Microsoft 365 (Microsoft, Redmond, WA, USA), IBM SPSS Statistics version 30 (IBM, Armonk, NY, USA), and JMP 19 (SAS Institute Inc., Cary, NC, USA) were used. The significance level was set to 5% (two-tailed). Bacteria with an average relative abundance lower than 1% and metabolites not detected in at least 75% of samples were excluded from the analysis. Measurements outside the quantification range were replaced with pre-specified substitute values. No adjustment was made for multiple testing of multiple items and multiple time points.

## 3. Results and Discussion

### 3.1. In Vitoro Results of Human Fecal Microbiota Culture Assay

**Figure 2:**
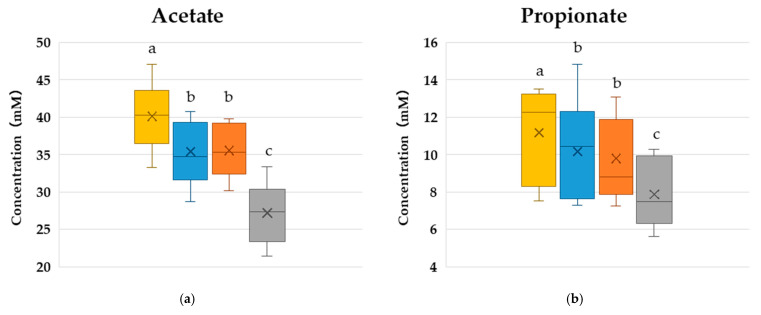

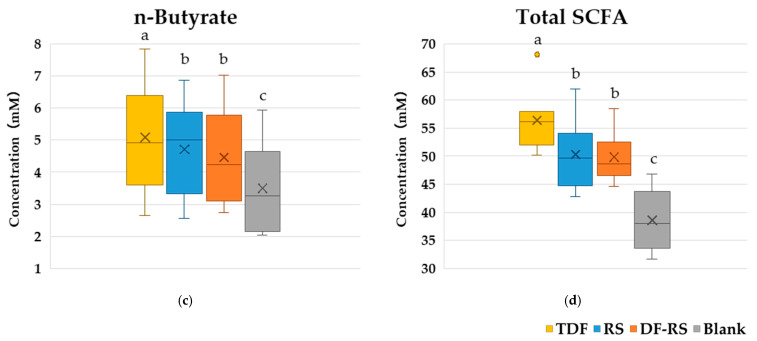
The effects of HAW flour fractions on SCFA production. (**a**) Acetate concentration. (**b**) Propionate concentration. (**c**) n-Butyrate concentration. (**d**) Total SCFA concentration (sum of acetate, propionate, and n-butyrate). Boxes represent the interquartile range (IQR), the horizontal line indicates the median, the whiskers indicate 1.5 × IQR, the cross (×) indicates the mean, and small circles indicate outliers. Significant differences between groups, represented with different letters (*p* < 0.05), were determined using the Wilcoxon signed-rank test (*n* = 9).

To evaluate the intestinal fermentability of the dietary fiber in HAW flour and explore the fermentation mechanisms, the TDF, RS, and DF-RS fractions were subjected to in vitro fecal fermentation, and their effects on SCFA production and the gut microbiota after culture were compared, as presented in Figure 2. Both DF-RS and RS significantly increased the production of all SCFAs (acetic acid, propionic acid, and n-butyric acid) compared with the effects of the blank control, indicating that these fractions are fermentable substrates for intestinal bacteria. Furthermore, TDF yielded greater SCFA production than either DF-RS or RS, suggesting that multiple fermentable dietary fibers in HAW flour were used additively or synergistically as substrates for intestinal bacteria. HAW flour contains more than 10 times more RS than regular wheat flour [13], alongside a greater variety of dietary fibers. Diverse dietary fibers can enhance intestinal microbiota diversity [35], and increased intestinal microbiota diversity is associated with multilayered SCFA production pathways, contributing to more stable SCFA production [36].

These results indicate that HAW might have greater potential to improve the gut environment through enhancing SCFA production than conventional wheat flour, as it contains RS in addition to other fermentable dietary fibers.

**Figure 3:**
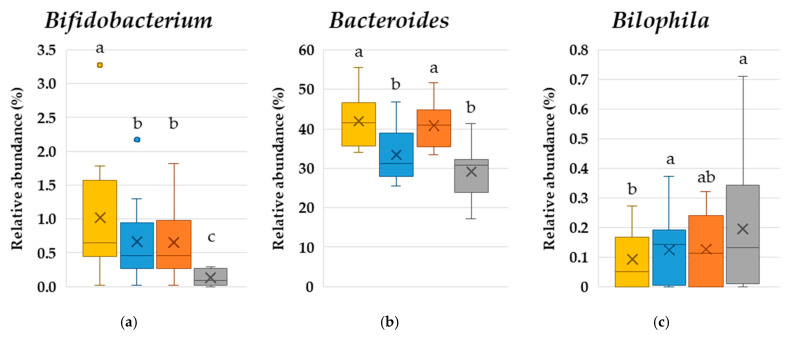

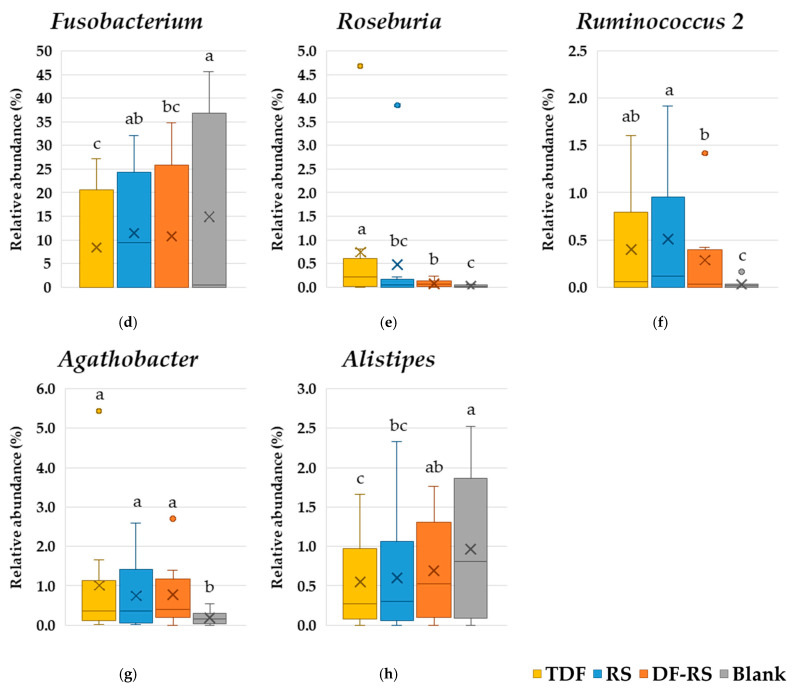
The effects of HAW flour fractions on the relative abundance of intestinal bacterial genera. The measured bacterial species followed MGScreening™. (**a**) *Bifidobacterium*. (**b**) *Bacteroides*. (**c**) *Bilophila*. (**d**) *Fusobacterium*. (**e**) *Roseburia*. (**f**) *Ruminococcus 2*. (**g**) *Agathobacter*. (**h**) *Alistipes.* Boxes represent the IQR, the horizontal line indicates the median, the whiskers indicate 1.5 × IQR, the cross (×) indicates the mean, and small circles indicate outliers. Significant differences between groups, represented with different letters (*p* < 0.05), were determined using Wilcoxon’s signed-rank test (*n* = 9).

The intestinal microbiota was also analyzed after culture, and the relative abundance of each genus was calculated according to the total number of bacteria. In both the DF-RS and RS groups, the relative abundance of *Bifidobacterium*, *Roseburia*, *Ruminococcus 2*, and *Agathobacter* was increased compared with that in the blank control, whereas the abundance of *Fusobacterium* and *Alistipes* was lower in the treatment groups (Figure 3). *Bifidobacterium* is a lactic acid- and acetic acid-producing bacterium, and *Roseburia* and *Agathobacter* are major butyric acid-producing bacteria [37]. Meanwhile, *Bilophila*, *Fusobacterium*, and *Alistipes* have been positively associated with diseases such as inflammatory bowel disease and colorectal cancer [38,39,40]. Furthermore, *Fusobacterium* and *Alistipes* promote the catabolism of proteins and amino acids and possess metabolic pathways for producing intestinal putrefactive products such as indole and p-cresol [41]. Thus, reductions in the abundance of these bacteria suggest a potential decrease in the production of putrefactive metabolites, consequently improving the intestinal environment. These results indicate that RS and DF-RS act as beneficial prebiotics by enhancing the abundance of SCFA-producing bacteria and suppressing the abundance of potentially harmful bacteria. In the TDF group, the abundance of *Bifidobacterium* and *Roseburia* was significantly increased, whereas the abundance of three harmful bacteria tended to be lower than that in the RS and DF-RS groups. These results suggest that HAW flour, which contains multiple fermentable dietary fibers including RS, exerts a stronger prebiotic effect than conventional wheat flour.

Among the aforementioned intestinal bacteria, only *Ruminococcus 2* and *Bacteroides* displayed differences in abundance between the RS and DF-RS groups. *Ruminococcus 2* has been reported to act as a primary degrader of RS [42], consistent with its remarkably higher abundance in the RS group in this study. *Ruminococcus 2* possesses an amylosome, an enzyme complex that degrades starch and produces oligosaccharides and glucose, on its cell surface [43]. These degradation products are believed to have supported the growth of *Ruminococcus 2* by allowing the utilization of RS in HAW flour. Previous studies reported that the abundance of *Ruminococcus 2* has a strong positive correlation with butyrate-producing bacteria such as *Roseburia* and *Agathobacter* in in vitro fermentation models [44], and co-cultivation of *Ruminococcus 2* and *Agathobacter* under RS-supplemented conditions significantly increased the abundance of *Agathobacter* [42]. This is believed to be because butyric acid bacteria utilized oligosaccharides and glucose derived from RS released by *Ruminococcus 2*, leading to greater abundance of these bacteria. In agreement with these findings, the results suggest that oligosaccharides and glucose derived from RS following decomposition by *Ruminococcus 2* were utilized by *Bifidobacterium*, *Roseburia*, and *Agathobacter*, promoting their growth and contributing to increased SCFA production. The elevation of SCFA levels is expected to reduce pH, leading to a reduction in harmful bacteria such as *Fusobacterium*, *Bilophila*, and *Alistipes*. Moreover, *Agathobacter* is involved in arabinoxylan fermentation [45], and *Bifidobacterium* is reported to degrade arabinoxylan and fructan [46,47], which explains their higher levels in the DF-RS group.

Interestingly, the abundance of *Bacteroides* increased in the DF-RS group but not in the RS group. Many species of this genus are involved in SCFA production [48] and are capable of degrading arabinoxylan and fructan [49,50]. Conversely, *B. thetaiotaomicron* does not readily degrade RS [42]. These findings are consistent with the current results that the abundance of *Bacteroides* increased in the DF-RS group but not in the RS group. Furthermore, co-cultivation with *Ruminococcus 2* under RS-supplemented conditions was found to increase the abundance of *B. thetaiotaomicron*, which used the oligosaccharides and glucose derived from RS produced by *Ruminococcus 2* [51]. In other words, the presence of RS plays an important role in the increased abundance of SCFA-producing *Bacteroides*.

Overall, these findings suggest a mechanism by which primary RS-degrading bacteria such as *Ruminococcus 2* catabolize RS into oligosaccharides and glucose, which subsequently promote the growth of beneficial SCFA-producing bacteria. These beneficial bacteria then degrade other fibers such as fructan and arabinoxylan, further contributing to SCFA production. In this proposed mechanism, RS, which initially promotes the growth of beneficial bacteria, is considered extremely important. Therefore, HAW, which contains high levels of RS, is considered a suitable material for improving the intestinal environment.

### 3.2. Results of the Clinical Trial

#### 3.2.1. Test Foods

The HAW food contained 5.5 g of total dietary fiber, versus 0.7 g for the control food. The HAW food contained 2.9 g of RS, whereas the RS content of the control food was below the detection limit (Table 1). Moreover, the HAW food contained greater amounts of arabinoxylan, arabinogalactan, and fructan than the control food. Unlike the control food, the HAW food contained all dietary fiber fractions, including insoluble, soluble high-molecular-weight, and soluble low-molecular-weight dietary fiber. Soluble dietary fiber is rapidly fermented in the proximal colon, whereas insoluble dietary fiber is fermented over a long period up to the distal colon [52]. Because HAW contains similar amounts of insoluble and soluble dietary fiber, it is expected to represent a prebiotic that can maintain the overall colon environment by allowing dietary fibers to be fermented by intestinal bacteria across multiple regions of the colon.

#### 3.2.2. Participants

No changes in the study design occurred after the start of the trial, and no participants discontinued or withdrew during the study period. All randomized subjects completed the trial (ITT analysis subjects). No significant intergroup differences were observed concerning the participants’ characteristics (Table 2).

#### 3.2.3. Evaluation of Effectiveness

The results of the full analysis revealed no significant difference in the frequency of bowel movements between the groups during 2 weeks of test food consumption (Table 3). Bowel movement frequency exhibits considerable day-to-day variability, and it is strongly influenced by dietary habits such as total food intake and dietary fiber consumption. Therefore, the relatively short intervention period of 2 weeks in this study and the variability in subjects’ lifestyle habits might have masked the intervention’s effects.

Conversely, the fecal concentration of p-cresol, an intestinal putrefactive product, was significantly lower in the HAW food group than in the control food group (Table 4). A previous study in which participants consumed 11.1 g/day of RS also recorded a significant reduction in p-cresol levels [15]. p-Cresol is produced from tyrosine or phenylalanine by particular intestinal bacteria, including putrefactive and potentially harmful species such as *Clostridioides difficile*, and increases in its levels have been associated with various diseases such as uremia, colon cancer, cardiovascular diseases, and autism [53]. A study of healthy adults demonstrated that higher fecal p-cresol concentrations are associated with increased levels of intestinal proteolytic fermentation markers, such as elevated ammonium levels and reduced carbohydrate levels in feces, indicating an unfavorable metabolic state in the gut [54]. Therefore, a reduction in p-cresol concentrations may reflect an improvement in intestinal metabolic balance even in healthy individuals, and p-cresol considered a representative indicator of deteriorated intestinal conditions. Furthermore, a large population-based study involving 3641 participants reported that 4-cresol sulfate, a major circulating metabolite of p-cresol, is associated with multiple health-related phenotypes, including metabolic syndrome and several cardiometabolic parameters [55]. These findings suggest that even within non-diseased populations, p-cresol related metabolites might influence systemic metabolic status and may potentially serve as biomarkers linked to broader aspects of health. Based on these observations, the decrease in p-cresol levels following HAW flour consumption in this study indicates that HAW might exert beneficial effects on the intestinal environment in healthy individuals. RS comprised approximately 50% of the dietary fiber content in HAW food (Table 1). In addition, the in vitro fecal culture experiments suggested that RS is preferentially degraded and is consumed as a nutrient source by beneficial bacteria, indicating that RS is an important functional component contributing to the effects of HAW flour.

The relative abundance of intestinal microbiota at the genus level (>1%) was calculated (Table 5), revealing a significant increase in the abundance of *Agathobacter* and significant decreases in the abundance of *Alistipes* and *Eubacterium hallii* group. The other bacterial genera with a relative abundance of 1% or higher are listed in Table S1. The increased abundance of *Agathobacter*, a representative butyrate-producing bacterium, was consistent with the results of the culture test (Figure 2). Conversely, no significant increase in fecal butyrate levels was detected in the clinical test. One possible explanation is that most of the butyrate produced in the intestine is absorbed by the intestinal epithelium, resulting in no measurable increase in fecal butyrate concentrations. For *Alistipes*, both the in vitro and in vivo studies consistently revealed a decrease in its abundance following HAW consumption. *Alistipes* has been reported to have a positive correlation with fecal p-cresol levels [54], and it is known to have multiple pathways of protein and amino acid fermentation, representing putrefactive mechanisms [40]. Based on these characteristics, the decreased abundance of *Alistipes* might have contributed to the suppression of p-cresol production from tyrosine or phenylalanine. *E. hallii* is also a butyrate-producing bacterium [37]. The cause of its decreased abundance remains unclear, but it might have been outcompeted by other bacteria (e.g., *Agathobacter*) for shared resources, particularly acetate, a precursor of butyrate.

These findings suggest that HAW food intake increased the abundance of *Agathobacter* and promoted butyrate production in the intestines. These changes, along with the production of butyric acid, led to improvements in the intestinal environment, such as a decrease in pH. Consequently, it is considered that the decreased abundance of *Alistipes*, which is involved in p-cresol production, led to reduced p-cresol levels in feces.

No significant differences were observed in other secondary endpoints, including fecal SCFA levels. The lack of a significant increase in SCFA levels in this trial contradicts the in vitro test results. In this trial, participants exhibited individual variability in SCFA production. This is attributable to the presence of responders, whose SCFA levels increased, and non-responders, who exhibited little change. In previous studies, a significant increase in butyrate levels was demonstrated after 4 weeks of wheat-derived RS intake (11.1 g/day) [15]. In this study, RS was consumed at a lower level (2.9 g/day) and for a shorter duration (2 weeks), which might also explain the lack of statistically significant changes in fecal SCFA levels.

#### 3.2.4. Evaluation of Effectiveness (Subgroup Analysis)

Participant-level evaluation of the trial results suggested that the presence of both responders, who exhibited increased butyrate production upon HAW food intake, and non-responders, who did not exhibit such an increase. To identify factors that might explain this variability, an important perspective of personalized nutrition, subgroup analysis was conducted as a post hoc analysis. Participants exhibiting greater increases in butyrate production tended to have lower daily dietary fiber intake (correlation coefficient: HAW, −0.3; control, 0.1). Based on this observation, we focused on dietary fiber intake.

Lower daily intake of dietary fiber has been reported as a risk factor for various diseases. For example, individuals consuming ≤10.6 g/day fiber have a higher risk of dementia [56], and those consuming ≤9.6 g/day exhibit higher risks of various cancers, including colorectal cancer [57]. Additionally, individuals with low dietary fiber intake have lower SCFA levels in the gut and impaired gut barrier function compared with individuals with higher intake [58,59]. From these findings, it was considered that individuals with a dietary fiber intake of ≤9.6 g/day have a more deteriorated gut environment, even among healthy individuals. It was therefore hypothesized that individuals with lower habitual fiber intake would exhibit a more pronounced response to fermentable dietary fibers, including RS present in HAW flour. Accordingly, the subgroup analysis targeted subjects whose dietary fiber intake was ≤9.6 g/day before the start of the trial.

In total, 59 subjects were eligible for effective analysis (28 in the control food group and 31 in the HAW food group, Table S2). Concerning background characteristics, a significant between-group difference was only observed for height, but this parameter was not considered to influence the primary or secondary evaluation items.

Among participants with ≤9.6 g/day dietary fiber intake, bowel movement frequency at week 2 was higher in the control group than in the HAW group (Table S3). The reason for this difference is unclear, but it might be related to individual responses to certain components in the control food. Further studies should examine these ingredients alongside lifestyle factors such as water intake and physical activity.

The effect of HAW flour on butyrate production was also evaluated in individuals with lower dietary fiber intake. Fecal butyrate content per bowel movement was significantly higher in the HAW group than in the control group (1.4 ± 1.3 mmol vs. 0.8 ± 0.5 mmol, *p* < 0.05). Butyrate plays important physiological roles, including maintaining intestinal barrier function, suppressing the growth of harmful bacteria, and promoting regulatory T cell differentiation, which contributes to systemic immune regulation [60]. Furthermore, recent clinical studies have demonstrated that increased butyrate production is associated with improvements in gut microbial diversity and reduced systemic inflammation in healthy adults [61]. Therefore, increasing butyrate levels through HAW flour intake might help improve the gut environment and prevent various diseases.

Previous studies identified an inverse correlation between daily dietary fiber intake and butyrate production during prebiotic intake [62]. In other words, individuals with a lower daily intake of dietary fiber tend to have higher butyrate production when consuming prebiotics. This result is interpreted as reflecting the limited availability of fermentable substrates for the gut microbiota. Thus, individuals with low fiber intake might exhibit more pronounced responses because of substrate insufficiency. The current results appear to align with this concept. Participants with low dietary fiber intake have insufficient substrates for the gut microbiota, and butyrate production increases with HAW flour intake, resulting in clearer gut environment improvement. Additionally, the fecal p-cresol concentration was significantly lower in the HAW food group (2.7 ± 2.1 μmol/g dry feces) than in the control group (3.9 ± 1.8 μmol/g dry feces) in week 2 (*p* = 0.02), consistent with the results of the full analysis set.

From these findings, responders to HAW flour are characterized by lower habitual dietary fiber intake. HAW flour was found to potentially improve the gut environment and reduce disease risk by promoting butyrate production in individuals with low dietary fiber intake. HAW flour can be used as a staple food in the same manner as regular wheat flour, making it highly practical for incorporation into daily diets. With insufficient dietary fiber intake being a global problem, HAW flour might contribute to health improvement in individuals whose fiber intake is below recommended levels.

#### 3.2.5. Safety Assessment

During the study period, seven participants experienced a total of eight adverse events, but none was serious. All adverse events were judged to not be causally related to the study food by the principal investigator. Therefore, no adverse effects were reported.

### 3.3. Limitations

Several limitations should be considered. First, the in vitro fermentation experiments and in vivo human intervention study had different evaluation settings. Although the in vitro system is useful for assessing the fermentative capacity of the gut microbiota, it does not fully reflect host absorption and metabolism, and it might not directly translate to in vivo metabolic dynamics. Furthermore, the in vitro experiments evaluated relatively short-term responses, whereas the human study assessed the effects of intake over a 2-week period. This difference might also influence interpretation of the findings. Second, multiple comparison corrections were not applied in the microbiome and metabolite analyses. These analyses were exploratory in nature, aiming to capture a broad range of potential biological signals. Therefore, applying stringent correction methods could have increased the risk of overlooking meaningful changes. Accordingly, uncorrected *p*-values were reported, and the findings should be interpreted with caution due to the potential for false-positive results. Third, the intervention period of this study was relatively short (2 weeks). Although significant changes were observed for p-cresol, other indicators such as SCFA did not display significant changes. Short-term interventions might be insufficient to capture gradual or slower physiological shifts, highlighting the need for longer-term studies to evaluate more stable effects. Fourth, the subgroup analysis based on dietary fiber intake (≤9.6 g/day) was exploratory and not pre-specified in the study protocol. This cutoff was determined retrospectively to explore potential factors associated with responsiveness. Therefore, the findings might be subject to selection bias and possess limited generalizability; therefore, they should be interpreted with caution. Further validation in studies with predefined criteria and adequately powered designs is needed.

## 4. Conclusions

In this study, the effects of fermentable dietary fibers contained in HAW on the intestinal environment were examined using both an in vitro fecal fermentation assay and a randomized, double-blind, parallel-group trial. The in vitro assay demonstrated that both the RS and DF-RS fractions of HAW flour were fermented by the gut microbiota, leading to significant increases in SCFA production. Moreover, the TDF fraction, which contains both fractions, induced the production of higher SCFA levels than either other fraction alone, suggesting that combinations of multiple fermentable fibers can enhance microbial fermentation.

In the human trial, no significant differences in bowel movement frequency were observed; however, the concentration of the putrefactive metabolite p-cresol was significantly reduced in the HAW food group. Analysis of the gut microbiota revealed an increase in bacterial taxa associated with SCFA production, consistent with the effects observed in the in vitro assay. These findings suggest that the consumption of HAW flour might help regulate microbial metabolic balance and contribute to improvements in the intestinal environment.

Overall, HAW flour exhibits potential as a practical prebiotic ingredient that helps support healthier gut microbial metabolism. As HAW flour can be incorporated into daily diets similarly to conventional wheat flour, it offers an accessible modality to support a healthy intestinal environment, especially for people with insufficient dietary fiber intake.

## Figures and Tables

**Table 1:** Nutritional profiles of the test foods.

Content per Serving	Control Food	HAW Food
Weight (g)	61.4 ± 1.0	62.4 ± 1.0
Moisture (g)	10.1 ± 0.2	13.4 ± 0.2
Energy (kcal) ^(^^a)^	251.5 ± 0.6	239.8 ± 0.8
Protein (g)	4.1 ± 0.0	5.3 ± 0.0
Ash (g)	0.7 ± 0.0	0.8 ± 0.0
Fat (g)	10.0 ± 0.0	10.6 ± 0.0
Sodium (mg)	231.1 ± 1.2	240.2 ± 1.0
Carbohydrate (g)	36.5 ± 0.1	32.2 ± 0.1
Sugar (g)	35.8 ± 0.1	26.7 ± 0.2
Total dietary fiber (g) ^(b)^	0.7 ± 0.0	5.5 ± 0.1
Insoluble dietary fiber (g) ^(b)^	n.d.	2.5 ± 0.0
Soluble high-molecular-weight dietary fiber (g) ^(b)^	n.d.	1.3 ± 0.0
Soluble low-molecular-weight dietary fiber (g) ^(b)^	0.7 ± 0.0	1.7 ± 0.2
HAW flour (g)	-	28.0±0.4
RS (g) ^(c)^	n.d.	2.9 ± 0.0
Arabinoxylan (g) ^(d)^	n.d.	0.6 ± 0.0
Arabinogalactan (g) ^(e)^	n.d.	0.3 ± 0.0
Fructan (g) ^(f)^	0.4 ± 0.0	0.9 ± 0.0

Nutrition profile per scone. ^(a)^ Energy conversion factors: protein, 4; lipid, 9; sugars, 4; dietary fiber, 2. ^(b)^ AOAC 2011.25 method; ^(c)^ AOAC 2002.02 method; ^(d)^ Arabinoxylan = 0.88 × (arabinose + xylose − 0.7 × galactose) [31]; ^(e)^ Arabinogalactan = 0.89 × [arabinose − (arabinose in arabinoxylan/xylose in arabinoxylan) × xylose + galactose] [32]; ^(f)^ Fructan assay kit (K-FRUC, Megazyme). “n.d.” means not detected.

**Table 2:** Background factors of the participants.

	Unit	Control Food	HAW Food
Number of participants	-	38	38
Sex (male/female)	-	9/29	7/31
Age	years	47.4 ± 9.5	47.7 ± 9.6
Height	cm	161.4 ± 7.1	161.9 ± 8.7
Weight	kg	57.5 ± 8.8	55.7 ± 9.0
Bowel movements (a)	times/week	3.5 ± 0.7	3.5 ± 0.7
Dietary fiber intake (b)	g/day	7.7 ± 2.8	7.7 ± 2.4

Data are displayed as the mean ± standard deviation. (a) bowel movements during the first 2 weeks of the pre-observation period. (b) calculated using the BDHQ conducted at screening.

**Table 3:** Number of bowel movements in participants (primary endpoint).

	Control Food	HAW Food	*p*-Value
Baseline	3.6 ± 1.0	3.7 ± 1.0	0.65
Week 1	4.1 ± 1.1	4.4 ± 1.5	0.19
Week 2	4.8 ± 1.6	4.2 ± 1.3	0.08

Baseline, week before intake; week 1, days 1–7 of intake; week 2, days 8–14 of intake. Data are displayed as the mean ± standard deviation.

**Table 4:** Putrefactive products in feces.

	Unit	Time Point	Control Food	HAW Food	*p*-Value
p-Cresol	μmol/g dry feces	Baseline	3.9 ± 3.0	3.1 ± 1.8	0.22
		Week 2	4.1 ± 2.6	2.9 ± 2.2	0.03 *
Indole	μmol/g dry feces	Baseline	2.1 ± 0.7	2.0 ± 0.6	0.54
		Week 2	2.1 ± 0.6	1.9 ± 0.7	0.23

The concentration of putrefactive products in feces was calculated. Phenol, 4-ethylphenol, and skatole levels were below the lower limit of quantification in over 75% of the subjects, and thus, they were excluded from the analysis. Data are displayed as the mean ± standard deviation. *: *p* < 0.05.

**Table 5:** Relative abundance of selected bacterial genera in feces.

Genera	Unit	Change	Control Food	HAW Food	*p*-Value
*Agathobacter*	%	Δ2w	0.0 ± 1.3	1.0 ± 2.1	0.03 *
*Alistipes*	%	Δ2w	0.1 ± 1.0	−0.2 ± 1.6	0.04 *
*Eubacterium hallii* group	%	Δ2w	−0.1 ± 1.1	−0.2 ± 0.6	0.03 *

For genus-level bacteria with an average occupancy rate exceeding 1%, changes with significant differences are listed. Data are displayed as the mean ± standard deviation. *: *p* < 0.05 (Mann–Whitney U test).

## Data Availability

The data underlying this article cannot be shared publicly due to the need to protect the privacy of individuals who participated in the study. The data will be shared on reasonable request to the corresponding author.

## Supplementary Materials

The following supporting information can be downloaded at: https://www.mdpi.com/article/10.3390/microorganisms14040797/s1. Table S1: Relative abundance of bacterial genera in feces. Table S2: Background factors of the participants (subgroup analysis). Table S3: Number of bowel movements in subgroup participants.
