# Supplementary material for: In Vivo and In Vitro Effects of Fermentable Dietary Fiber from High-Amylose Wheat Containing Resistant Starch on the Intestinal Environment: A Randomized, Double-Blind, Placebo-Controlled, Human Trial
Source: Microorganisms. 2026 Apr 1;14(4):797. doi: 10.3390/microorganisms14040797 (PMC13119115; doi:10.3390/microorganisms14040797)
Supplement: Supplementary file 1 [file microorganisms-14-00797-s001.zip › microorganisms-4207940-supplementary.pdf]

Table S1. Relative abundance of bacteria genera in feces

Genera	Unit	Time point	Control food	HAW food	p-value
Bacteroides	%	0w	23.5 ± 11.6	25.5 ± 13.3	0.94
	%	2w	23.8 ± 10.1	27.6 ± 14.0	0.22
	%	Δ2w	0.3 ± 8.0	2.2 ± 8.6	0.42
Bifidobacterium	%	0w	12.3 ± 11.3	8.8 ± 7.2	0.33
	%	2w	11.3 ± 11.8	9.0 ± 8.5	0.38
	%	Δ2w	−0.1 ± 10.1	0.2 ± 5.9	0.35
Blautia	%	0w	8.4 ± 3.8	7.9 ± 4.0	0.60
	%	2w	8.4 ± 3.0	7.3 ± 4.3	0.11
	%	Δ2w	0.0 ± 3.6	−0.6 ± 3.1	0.28
Faecalibacterium	%	0w	6.3 ± 4.3	6.6 ± 5.0	0.89
	%	2w	6.6 ± 4.7	6.3 ± 4.8	0.74
	%	Δ2w	0.4 ± 4.1	−0.3 ± 3.6	0.89
Prevotella 9	%	0w	2.2 ± 7.0	3.6 ± 10.4	0.72
	%	2w	1.6 ± 5.2	4.8 ± 12.8	0.60
	%	Δ2w	−0.6 ± 6.6	1.2 ± 5.1	0.28
Fusicatenibacter	%	0w	2.8 ± 2.8	3.1 ± 3.2	0.72
	%	2w	3.1 ± 3.5	2.6 ± 2.3	0.97
	%	Δ2w	0.3 ± 1.7	−0.5 ± 2.5	0.30
Agathobacter	%	0w	1.6 ± 2.5	3.3 ± 5.2	0.44
	%	2w	1.7 ± 2.4	4.3 ± 5.2	0.08
	%	Δ2w	0.0 ± 1.3	1.0 ± 2.1	0.03*
Anaerostipes	%	0w	2.8 ± 2.2	2.6 ± 1.9	0.61
	%	2w	2.7 ± 2.6	2.2 ± 2.0	0.19
	%	Δ2w	-0.1 ± 1.8	−0.5 ± 1.5	0.21
Parabacteroides	%	0w	2.5 ± 1.9	2.5 ± 2.2	0.82
	%	2w	2.6 ± 2.3	2.0 ± 1.4	0.56
	%	Δ2w	0.1 ± 1.7	−0.5 ± 1.50	0.24
Ruminococcus torques group	%	0w	1.7 ± 1.8	1.4 ± 1.4	0.46
	%	2w	2.0 ± 2.0	1.1 ± 1.0	0.10
	%	Δ2w	0.3 ± 1.4	−0.3 ± 1.0	0.10
Collinsella	%	0w	1.8 ± 1.7	1.4 ± 1.2	0.38
	%	2w	1.5 ± 1.1	1.2 ± 1.1	0.13
	%	Δ2w	−0.2 ± 1.3	−0.2 ± 0.7	0.23
Alistipes	%	0w	1.4 ± 1.8	1.5 ± 1.6	0.42
	%	2w	1.5 ± 1.7	1.3 ± 1.4	0.88
	%	Δ2w	0.1 ± 1.0	−0.2 ± 1.6	0.04*
Lachnoclostridium	%	0w	1.2 ± 1.1	1.6 ± 1.1	0.09
	%	2w	1.3 ± 0.9	1.7 ± 2.1	0.94
	%	Δ2w	0.1 ± 0.7	0.1 ± 1.7	0.09
Eubacterium hallii group	%	0w	1.6 ± 1.4	1.2 ± 1.2	0.24
	%	2w	1.4 ± 0.9	1.0 ± 1.1	0.02*
	%	Δ2w	−0.1 ± 1.1	−0.2 ± 0.6	0.03*
Megamonas	%	0w	1.2 ± 4.5	0.9 ± 3.2	0.06
	%	2w	1.9 ± 6.4	0.7 ± 2.9	0.15
	%	Δ2w	0.8 ± 5.5	−0.2 ± 1.7	0.95
Subdoligranulum	%	0w	1.5 ± 1.5	0.8 ± 1.1	0.02*
	%	2w	1.4 ± 1.4	0.9 ± 1.2	0.08
	%	Δ2w	−0.1 ± 1.0	0.1 ± 0.5	0.96

Roseburia	%	0w	1.3 ± 4.1	1.2 ± 1.3	0.41
	%	2w	0.8 ± 1.1	1.2 ± 1.7	0.89
	%	Δ2w	−0.4 ± 4.4	0.0 ± 1.3	0.37
Ruminococcus gnavus group	%	0w	0.8 ± 1.4	1.1 ± 1.9	0.76
	%	2w	0.9 ± 1.0	1.3 ± 2.2	0.74
	%	Δ2w	0.1 ± 0.9	0.1 ± 1.0	0.62

For genus-level bacteria with an average occupancy rate exceeding 1%, changes with significant differences are listed. Data are displayed as the mean ± standard deviation. *: $p < 0.05$ (Mann–Whit- ney U test)

Table S2. Background factors of the participants (subgroup analysis)

	Unit	Control food	HAW food
Number of participants	-	28	31
Sex (male/female)	-	3/25	6/25
Age	years	46.8±9.5	47.5±9.8
Height*	cm	159.8±7.9	163.9±7.4
Weight	kg	55.4±6.6	55.8±9.5
Bowel movements a)	times/week	3.5±0.7	3.5±0.7
Dietary fiber intake b)	g/day	6.3±1.3	6.9±1.6

Data are displayed as the mean ± standard deviation. a) Bowel movements during the first 2 weeks of the pre-observation period. b) Calculated using the BDHQ conducted at screening. p*<0.05

Table S3. Number of bowel movements in subgroup participants

	Control food	HAW food	p-value
Baseline	3.5±1.0	3.7±0.8	0.31
Week 1	4.2±1.2	4.6±1.4	0.23
Week 2	5.2±1.6	4.3±1.2	0.02*

Baseline, week before intake; week 1, days 1–7 of intake; week 2, days 8–14 of intake. Data are displayed as the mean ± standard deviation.
